# Iron Overload Accelerates Aging-Associated Kidney Injury in Mice: Implications for Iron Supplementation in the Elderly

**DOI:** 10.3390/nu17162580

**Published:** 2025-08-08

**Authors:** Mungunchimeg Chultemsuren, Soo-Jin Song, Ki-Hwan Han, Jung-A Shin

**Affiliations:** Department of Anatomy, Ewha Womans University College of Medicine, Seoul 07804, Republic of Korea; mongoo0920@gmail.com (M.C.); sjsong9@gmail.com (S.-J.S.); khhan@ewha.ac.kr (K.-H.H.)

**Keywords:** kidney, iron overload, aging, glomerulus

## Abstract

**Background/Objectives:** Although essential for oxygen transport and DNA synthesis, excess iron is toxic and can damage organs such as the kidneys. Research has shown that iron overload induces kidney injury, and aging contributes to kidney dysfunction through functional and structural changes. The interaction between iron overload and aging remains poorly understood. Therefore, this study investigated their combined effects on renal microstructure and function using an iron-dextran-injected mouse model. **Methods:** Young and old mice were divided into control and iron overload groups, renal function was evaluated by serum creatinine and albuminuria, and urinary iron excretion was also measured to assess iron handling. The structural changes were assessed using histological analysis and electron microscopy. **Results:** Although the iron overload groups had similar blood iron levels, the old iron overload group exhibited significantly higher levels of albuminuria, urinary iron excretion, and serum creatinine compared with the young group. In the iron overload model, histological and ultrastructural analyses demonstrated iron accumulation in mesangial and endothelial cells, glomerular basement membrane thickening, and foot process widening, which were more pronounced in aged mice, suggesting that aging exacerbates iron-induced kidney injury. **Conclusions:** These findings demonstrate that aging increases susceptibility to iron-induced kidney injury, as shown by the accelerated glomerular injury observed in iron-overloaded aged mice. Therefore, elucidating the effects of aging on iron metabolism may contribute to identifying approaches for reducing age-associated renal injury.

## 1. Introduction

Iron is vital for all living organisms as it plays important roles in various processes, including oxygen transport, DNA synthesis, and electron transfer [1,2,3]. Because iron is essential for producing hemoglobin, iron supplements are the primary treatment for anemia, a common condition worldwide [4,5,6]. However, iron levels in the body must be carefully managed to prevent overload, which can occur in conditions such as hereditary hemochromatosis, frequent blood transfusions, or acute iron poisoning [4,5,6,7]. Excessive iron consumption can negatively affect various tissues and organs, including the liver, heart, and kidneys [5,8,9].

The kidneys filter waste products and excess substances from the blood, thereby maintaining fluid and electrolyte balance in the body [10,11]. The kidney’s primary filtration unit is the glomerulus, a highly specialized capillary network [12,13]. Blood filtration occurs through the glomerular filtration barrier, which comprises three layers: fenestrated endothelial cells, glomerular basement membrane (GBM), and podocytes with interdigitating foot processes. This barrier selectively permits the passage of water, electrolytes, and small solutes into the renal tubules while preventing the loss of large molecules, specifically albumin. After filtration, the molecules in the glomerular filtrate undergo selective reabsorption and secretion in the renal tubules to maintain homeostasis. Therefore, any disruptions of this filtration barrier can lead to proteinuria and impaired kidney function [14,15,16].

Iron follows a similar process, as filtered iron is primarily reabsorbed in the proximal tubules to regulate the systemic iron balance [17]. Circulating iron is primarily bound to transferrin (TF). However, under physiological conditions, small amounts of transferrin-bound iron (TBI) are filtered by the glomerulus [18,19]. Previous studies suggest that the proximal tubules may regulate renal iron homeostasis by reabsorbing filtered TBI through transferrin receptor 1 and the cubilin–megalin complex [17,20,21]. Iron export via ferroportin in proximal tubule cells is thought to be regulated by hepcidin, although its exact role in renal physiology is still under investigation [22,23]. Distal tubules appear to lack ferroportin and may play a lesser role in iron handling [24,25].

Previous studies have reported that iron overload affects glomerular and tubular structures, potentially leading to structural and functional alterations in the kidney [9,19,26,27]. Prolonged exposure to or high doses of iron have been shown to cause iron accumulation in the glomeruli, leading to focal thickening of the GBM and effacement of the podocyte foot process. These changes may compromise the filtration barrier’s integrity, thereby increasing glomerular permeability. Mesangial cell deposition, another key component of the glomerulus, is particularly prominent because mesangial cells actively take up iron through endocytosis and store it in phagolysosomes [28,29]. In the proximal tubule, iron overload induces oxidative stress, inflammation, and apoptosis, leading to cellular injury and impaired tubular function [24].

In addition to iron overload, aging is a significant factor in kidney deterioration as it drives structural and functional decline. Previous studies have observed a progressive reduction in glomerular number and size with aging, often accompanied by age-related glomerulosclerosis and proximal tubular atrophy [30,31]. These structural changes are associated with a decline in the glomerular filtration rate [32,33,34]. Although previous studies have investigated iron overload in younger subjects and explored the effects of normal aging, the interplay between these two factors remains unclear.

The present study aimed to examine the impact of iron overload on the renal microstructure and function in aging using an iron-dextran-injected mouse model. The renal function of mice was evaluated by measuring serum creatinine and albuminuria. In addition, urinary iron excretion was measured to evaluate renal iron handling. The structural changes were analyzed through histological and ultrastructural analyses. Our findings emphasize the combined effects of aging and iron overload on renal impairment, focusing on glomerular filtration rate and albuminuria.

## 2. Materials and Methods

### 2.1. Animals

Female C57BL/6J mice were used in this study. Old mice were obtained from the Korea Research Institute of Bioscience and Biotechnology (Daejeon, Republic of Korea), whereas young mice were acquired from Orient Bio (Seongnam-si, Republic of Korea). All mice were specific-pathogen-free (SPF), non-genetically modified, and had not undergone any previous experimental procedures. Mice were randomly assigned to four groups (n = 30 per group): young control (2 months), old control (22 months), young iron overload, and old iron overload. In the animal experiments, iron overload was induced by intraperitoneal injection of iron dextran (Sigma-Aldrich, MO, US; iron content 95–105 mg/mL), which was diluted in 0.9% NaCl to a final concentration of 10 mg/mL elemental iron. Mice received injections at a dose of 500 mg/kg body weight (elemental iron), five times per week for four consecutive weeks [35,36,37,38,39]. The control group received 0.9% saline, while the iron-overloaded group received iron dextran at the same dosage and schedule. The injections were performed at the same time each day.

Before sacrifice, the mice were placed in metabolic cages with free access to food and water, and urine collected for 19 h was obtained. Urinary albumin levels were measured using the Cobas c702 analyzer (Roche Diagnostics, Basel, Switzerland) with the ALBT2 reagent kit (Cat. No. 5167043190), according to the manufacturer’s instructions. Urinary iron levels were measured by a laboratory (SCL Healthcare Central Laboratory, Gyeonggi-do, Korea) using a colorimetric assay with the IRON2 reagent on the Cobas c 702 module (Roche Diagnostics, Basel, Switzerland) (n = 4). Albuminuria and urinary iron excretion were evaluated using total urine collected over a 19 h collection period. The mice were anesthetized using 20% isoflurane in propylene glycol solution (Samchun, Seoul, Korea). Blood samples were collected from the heart before sacrifice. The blood was then separated into serum by centrifugation for 20 min at 4 °C. The serum iron level, total iron-binding capacity (TIBC), unsaturated iron-binding capacity (UIBC), and TF saturation were measured using the Pointe Scientific Iron/TIBC Reagent Kit (MedTest Dx, Canton, MI, USA). Serum creatinine levels were measured using the DetectX^®^ Serum Creatinine Detection Kit (KB02-H, Arbor Assays, Ann Arbor, MI, USA), which is based on a modified Jaffe colorimetric method. Optical density was measured at 490 nm using a microplate reader according to the manufacturer’s instructions (n = 5).

This study was approved by the Institutional Animal Care and Use Committee of Ewha Womans University College of Medicine (EWHA MEDIACUC 21-003-8).

### 2.2. Tissue Preparation

The kidneys were perfused through the heart with phosphate-buffered saline (PBS, pH 7.4) for 3 min, followed by fixation with 4% paraformaldehyde [40]. The kidneys were then trimmed into 2–3 mm pieces and immersed in the same fixative at 4 °C overnight. Following dehydration through an alcohol series, the tissues were embedded in paraffin wax (Leica Biosystems, Wetzlar, Germany). The paraffin-embedded tissues were cut into 3-µm-thick sections (n = 8).

### 2.3. Perls’ Prussian Blue Staining

Perls’ Prussian Blue staining detects ferric iron in tissues, resulting in the formation of a blue color. The sections were deparaffinized in xylene and dehydrated through an ethanol series. After washing with distilled water, the sections were incubated for 30 min in a mixture of equal parts of 20% HCl and 10% potassium ferrocyanide and incubated for 30 min. After rinsing in tap water, the sections were stained with neutral fast red. Thereafter, they were dehydrated in ethanol and cleared with xylene, mounted with Permount (Thermo Fisher Scientific, Waltham, MA, USA), and observed using an Olympus BX50 light microscope. Perls’ Prussian-blue-stained areas were measured using ImageJ software version 1.53 (NIH, Bethesda, MD, USA), with at least five 40× images acquired from each of the three groups.

### 2.4. Periodic Acid–Schiff (PAS) Stain

The tissue sections were deparaffinized and rehydrated with water. Afterward, they were incubated in a 0.5% periodic acid solution for 5 min. After washing with DW, the sections were immersed in Schiff’s reagent (Sigma-Aldrich, St. Louis, MO, USA) for 15 min. Subsequently, the sections were thoroughly washed with tap water. Then, the sections were counterstained with hematoxylin. Finally, the sections were dehydrated in ethanol series, cleared with xylene, and mounted with Permount. PAS staining was used to score tubular injury in 30 selected tubules from kidney sections using a scale of 0 to 4 [41,42]. Tubular injury was semi-quantitatively scored on a scale of 0 to 3 as follows: 0, normal tubules without injury; 1, mild focal dilatation with occasional epithelial attenuation; 2, patchy moderate dilatation with more extensive attenuation, mild vacuolization, and edema; and 3, marked dilatation with diffuse epithelial attenuation, vacuolization, and edema.

### 2.5. Masson’s Trichrome Staining

Masson’s trichrome staining was performed using a kit (KH07007, Bioquochem, Oviedo, Spain). Paraffin sections were deparaffinized, hydrated, and fixed in Bovin fixative at 60 °C for 60 min. After rinsing, nuclei were stained with Weigert’s hematoxylin for 5 min. Biebrich Scarlet was applied for 15 min, followed by phosphotungstic–phosphomolybdic acid for 15 min and aniline blue for 10 min. The slides were placed in 1% acetic acid for 5 min, then dehydrated in ethanol, cleared with xylene, and mounted. Three slides per group were selected, and three images were captured from each slide for analysis using ImageJ.

### 2.6. Transmission Electron Microscopy (TEM)

Kidney tissue containing the cortex was sectioned into 1 mm × 1 mm pieces and examined using an electron microscope as previously described [43]. In summary, the kidney tissue was fixed in 2.5% glutaraldehyde and then embedded in epoxy resin (Epon 812, Sigma-Aldrich, St. Louis, MO, USA). Next, 70–80-nm-thick sections were prepared using a diamond knife and an ultramicrotome. The sections were then stained with 10% uranyl acetate and examined using a transmission electron microscope (H-7650, Hitachi, Tokyo, Japan). The thickness of the GBM was measured from 30 randomly selected GBMs, and the width of the foot process was measured from 60–80 processes in TEM images at 10,000× magnification. All measurements were performed using ImageJ software.

### 2.7. Statistical Analysis

Statistical analyses were performed using unpaired two-tailed t-tests to determine differences between groups. Graphs were created using GraphPad Prism 8.0 (San Diego, CA, USA). Data are expressed as mean ± standard deviation (SD). A *p*-value < 0.05 was considered statistically significant. This study was not blinded.

## 3. Results

### 3.1. Similar Increases in Serum Iron Markers Between the Young and Old Iron Overload Groups

**Figure 1:**
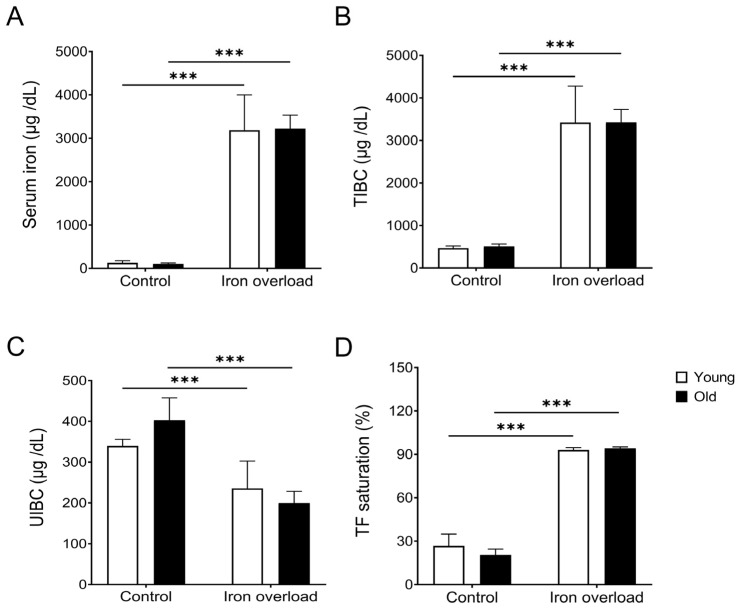
Blood iron analysis in the control and iron overload groups. The serum iron levels were significantly higher in the iron overload groups than in the control groups (**A**). TIBC was markedly increased in the iron overload groups than in the control groups (**B**). The UIBC decreased significantly in the iron overload groups than in the control groups (**C**). The TF saturation was significantly elevated in the iron overload groups than in the control groups (**D**). Differences were significant at *** *p* < 0.001.

An analysis was performed to verify whether the iron overload model had been established. The measurements included serum iron level, TIBC, UIBC, and TF saturation (Figure 1). The serum iron levels were significantly higher in the young (3186.43 ± 812.87 vs. 127.42 ± 49.28 µg/dL, *p* < 0.001) and old iron overload groups (3224.51 ± 307.67 vs. 103.9 ± 19.76 µg/dL, *p* < 0.001) compared with their respective control groups (Figure 1A). The TIBC, which indicates the total amount of iron that TF can bind, was significantly increased in the young (3421.76 ± 855.31 µg/dL) and old iron overload groups (3424.37 ± 306.57 µg/dL) compared with the young (466.95 ± 51.07 µg/dL, *p* < 0.001) and old control groups (506.87 ± 58.90 µg/dL, *p* < 0.001) (Figure 1B). The UIBC, which represents the capacity of TF that has not yet bound to iron, was markedly decreased in the young iron overload group (235.33 ± 67.14 µg/dL) compared with the young control group (339.54 ± 16.43 µg/dL, *p* < 0.05) and significantly decreased in the old iron overload group (199.86 ± 28.55 µg/dL) compared with the old control group (402.97 ± 54.39 µg/dL, *p* < 0.001) (Figure 1C). Meanwhile, the TF saturation was significantly higher in the young (92.99 ± 1.68% vs. 26.70 ± 8.18%, *p* < 0.001) and old iron overload groups (94.13 ± 0.98% vs. 20.58 ± 3.89%, *p* < 0.001) compared with their respective control groups (Figure 1D).

### 3.2. The Old Iron Overload Group Showed Increased Creatinine, Urinary Iron Excretion, and Albuminuria

**Figure 2:**
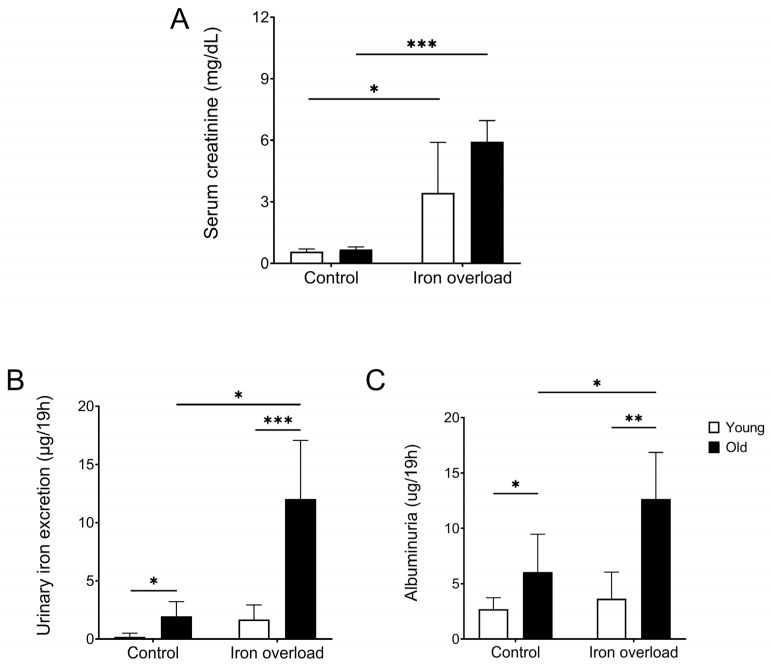
Comparative analysis of renal function biomarkers and urinary iron parameters. The serum creatinine levels were higher in the iron overload groups than in the control groups (**A**). The urinary iron excretion was elevated in the iron overload groups compared with their respective controls, with the old iron overload group exhibiting the highest levels (**B**). Albuminuria increased with age, and the old iron overload group showed higher levels of albuminuria than the old control group (**C**). Differences were significant at * *p* < 0.05, ** *p* < 0.01, and *** *p* < 0.001.

An analysis was performed to investigate the effects of iron overload and aging on kidney function and related parameters. Kidney function was assessed by measuring the creatinine levels (Figure 2A). The results showed that the serum creatinine levels were significantly increased in the young (3.44 ± 2.47 mg/dL, *p* < 0.05) and old iron overload groups (5.93 ± 1.03 mg/dL, *p* < 0.01) compared with the young (0.57 ± 0.13 mg/dL) and old control groups (0.68 ± 0.11 mg/dL).

Urinary iron excretion was measured to evaluate renal iron handling and assess potential iron-overload-related kidney dysfunction (Figure 2B). The urinary iron excretion in the old control group (1.95 ± 1.26 µg) was higher than that in the young control group (0.18 ± 0.32 µg, *p* < 0.05). Moreover, the urinary iron excretion in the old iron overload group (12.02 ± 5.04 µg) was higher than that in the young iron overload group (1.68 ± 1.24 µg, *p* < 0.01). Furthermore, the old iron overload group showed a significant increase in urinary iron excretion compared with the old control group (*p* < 0.01).

According to previous studies, proteinuria increases with aging [44,45]. This finding is consistent with our observation that the old control group (6.06 ± 1.56 μg) showed significantly higher albuminuria compared with the young control group (2.72 ± 0.42 μg, *p* < 0.05) (Figure 2C). Similarly, the young iron overload group (3.65 ± 0.88 μg) had lower albuminuria than the old iron overload group (12.65 ± 1.88 μg, *p* < 0.01). In addition, among the old groups, the old iron overload group exhibited significantly higher albuminuria compared with the old control group (*p* < 0.05).

### 3.3. Iron Deposition Increased in the Old Iron Overload Group than in Other Groups

**Figure 3:**
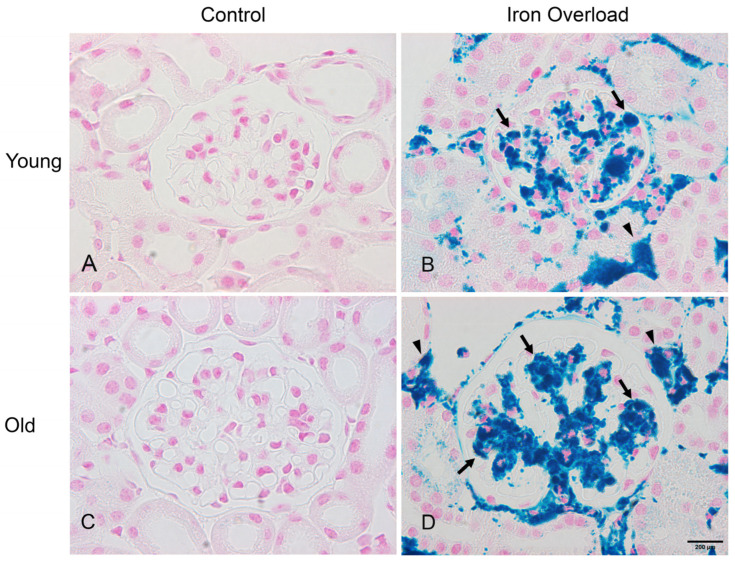
Iron deposition in the renal tissue of the young and old groups. No iron deposition was observed in the young and old control groups (**A**,**C**). Perl’s Prussian blue staining indicated that iron deposition was present in the glomerulus (arrows) and interstitium (arrowheads) of the young and old iron overload groups, with the old group showing more pronounced staining (**B**,**D**). Scale bar = 200 μm.

Perls’ Prussian blue staining detects iron in tissues by forming blue deposits, allowing for the assessment of iron distribution within the kidney (Figure 3). Iron deposition was not observed in the control groups (Figure 3A,C). By contrast, blue staining was present in the glomerulus (arrows) and interstitium (arrowheads) of the iron overload groups, suggesting increased iron deposition in these areas (Figure 3B,D). The measurement of Perls’ Prussian blue stain intensity revealed an increase in the young (16.02 ± 6.14) and old iron overload groups (22.22 ± 3.46) compared to the young (0.08 ± 0.06, *p* < 0.001) and old control groups (0.15 ± 0.14, *p* < 0.001). Moreover, the old iron overload group showed higher iron deposition than the young iron overload group (*p* < 0.01).

### 3.4. Renal Structural Changes Were Frequently Observed in the Iron Overload Aged Group

**Figure 4:**
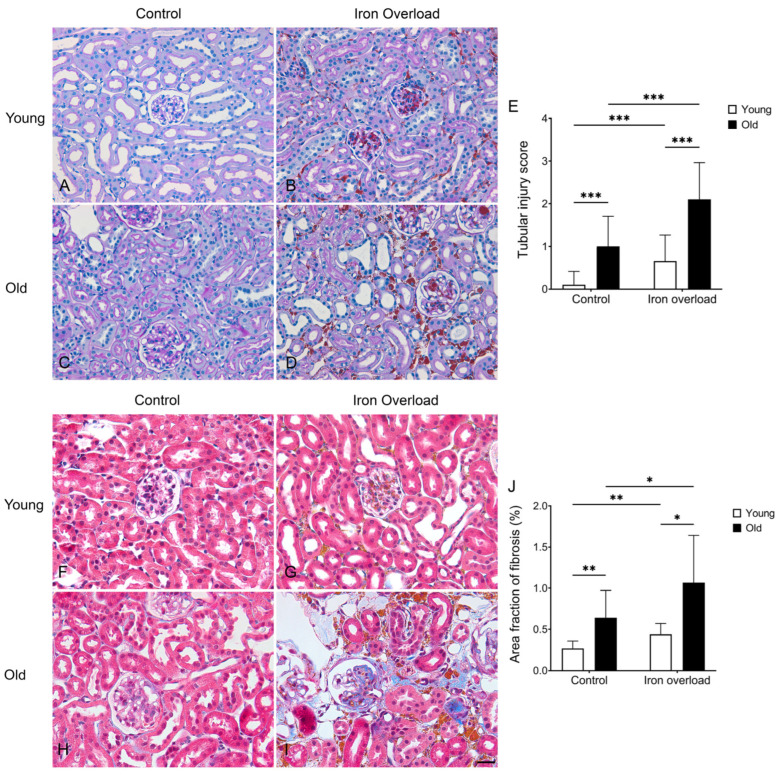
PAS staining (**A**–**D**) and Masson’s Trichrome staining (**F**–**I**) of mouse kidney tissue following iron overload. The young control group exhibited a normal glomerulus (**A**). Iron deposition was observed in the mesangial area of the iron overload groups (**B**). The old control group showed luminal dilation in the glomerular capillaries (**C**). However, the old iron overload group showed more pronounced dilation and iron deposition (**D**). The tubular injury score was observed in the control old group and the iron overload young group, with the most severe injury in the iron overload old group (**E**). Masson’s Trichrome staining of young control mice showed normal kidney structure (**F**). Young iron-overloaded mice had slight collagen accumulation in the interstitium (**G**). Aged control mice showed interstitial fibrosis due to aging (**H**). Aged iron-overloaded mice showed more pronounced fibrosis, with greater collagen deposition (**I**). Area fraction of fibrosis showed increased fibrosis in the control old and the iron overload young group, and with the iron overload old group showing the greatest amount of fibrosis (**J**). Difference was significant at * *p* < 0.05, ** *p* < 0.01, and *** *p* < 0.001. Scale bar = 400 μm in (**A**–**D**), 200 μm in (**F**–**I**).

PAS staining was performed to evaluate the extent of structural changes in the mouse kidney following iron overload (Figure 4). The young control group showed a normal glomerulus (Figure 4A). Meanwhile, the iron overload groups showed a yellowish-brown deposition in the glomerulus and interstitium, with the old iron overload group showing more extensive accumulation compared with the young iron overload group (Figure 4B,D). Similar to previous studies [46,47], the glomerular capillaries in the old control group showed luminal dilation (Figure 4C). The old iron overload group exhibited even more severe capillary dilation compared with the old control group (Figure 4D). Tubular injury was scored from 0 to 4 as described in the Materials and Methods, and the control young group (0.10 ± 0.06) showed minimal tubular injury with no significant alterations. In contrast, tubular injury was observed in the control old group (1.00 ± 0.13, *p* < 0.001) and the iron overload young group (0.66 ± 0.11, *p* < 0.001), with the most pronounced injury occurring in the iron overload old group (2.10 ± 0.16, *p* < 0.001) (Figure 4E).

### 3.5. Renal Fibrosis Was Markedly Exacerbated in Aged Mice Under Iron Overload Conditions

Masson’s trichrome staining was performed to evaluate collagen deposition in the kidney (Figure 4). In young iron-overloaded mice, slight collagen accumulation was observed in the interstitium (Figure 4G). Aged control mice exhibited interstitial fibrosis, which is likely related to age-associated structural changes (Figure 4H) [48,49]. In contrast, aged mice with iron overload showed more pronounced fibrosis, characterized by increased collagen deposition (Figure 4I). Collagen deposition was measured to determine the impact of aging and iron overload (Figure 4J). It was significantly higher in the control old group (0.64 ± 0.33%) than in the control young group (0.27 ± 0.09%, *p* < 0.01) and in the iron overload old group (1.06 ± 0.57%) than in the iron overload young group (0.44 ± 0.12%, *p* < 0.05). The iron overload young group also showed increased fibrosis compared to the control young group (*p* < 0.01), and the iron overload old group showed a further increase compared to the control old group (*p* < 0.05). These results suggest that aging and iron overload may contribute to increased kidney fibrosis.

### 3.6. Greater Iron Deposition in Renal Mesangial Cells Was Observed in the Old Iron Overload Group

**Figure 5:**
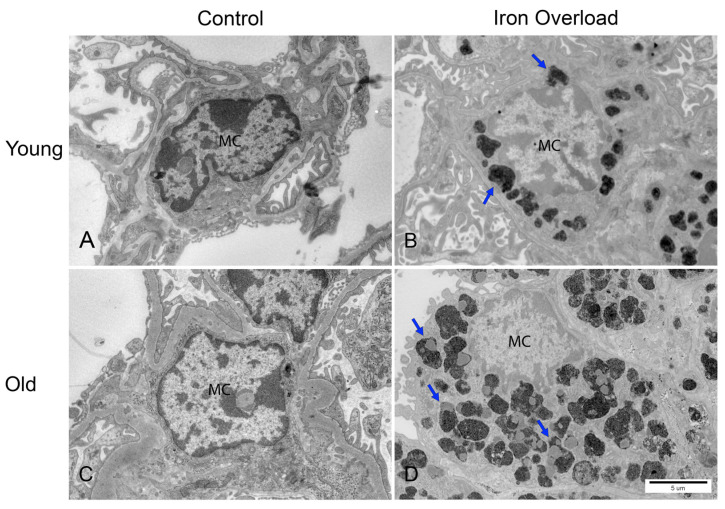
TEM analysis of mouse kidney mesangial cells. Normal mesangial cells within the glomerular tuft (**A**,**C**). Iron deposition in mesangial cells (arrows), with more pronounced accumulation in the old iron overload group (**B**,**D**). Enlarged mesangial cells containing phagolysosomes with electron-dense granules are observed in the old iron overload group (**D**). MC, mesangial cell. Scale bar = 5 μm.

Under TEM, mesangial cells were identified within the glomerular tuft, typically located between capillary loops, exhibiting an irregular shape with a densely packed nucleus [14] (Figure 5). In the iron overload groups, iron deposition was observed in the mesangial cells. This observation is consistent with the findings of previous studies, which demonstrated similar iron accumulation in the experimental models of the young iron overload group [28,29] (Figure 5B). The accumulation appeared to be more pronounced in the old iron overload group than in the young iron overload group (Figure 5B,D). In the iron overload groups, the mesangial cells appeared to be enlarged and contained phagolysosomes with dark, electron-dense deposits, suggesting intracellular iron accumulation (Figure 5D, arrows).

### 3.7. Iron Accumulation Was Increased in Aged Glomerular Endothelial Cells

**Figure 6:**
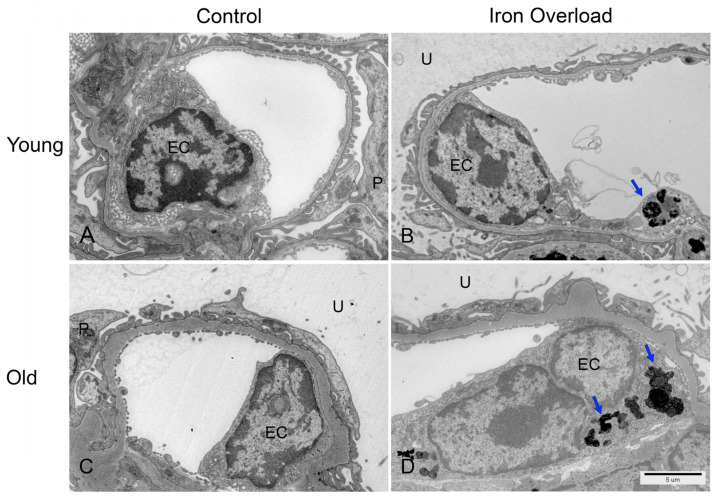
TEM images of glomerular endothelial cells in the control and iron overload groups. Normal glomerular endothelial cells in the control group (**A**,**C**). Electron-dense iron deposits (arrow) in the glomerular endothelial cells of the young iron overload group (**B**). More pronounced iron accumulation (arrow) in the glomerular endothelial cells of the old iron overload group (**D**). Podocyte (P), endothelial cells (EC), and urinary space (U). Scale bar = 5 μm.

The changes in glomerular endothelial cells following iron overload were examined using TEM (Figure 6). Kidney endothelial cells are characterized by fenestrations, a thin cytoplasm, and close contact with the GBM [50]. In the present study, iron deposition was not detected in the endothelial cells of the control groups (Figure 6A,C). According to previous studies, iron overload leads to light iron loading in glomerular endothelial cells [28]. Our findings in the young iron overload group are consistent with these observations (Figure 6B, arrows). TEM revealed electron-dense iron deposits in glomerular endothelial cells, with the old iron overload group showing slightly greater iron accumulation (Figure 6D, arrows).

### 3.8. GBM Thickening and Foot Process Widening Were More Significant in the Old Iron Overload Group

**Figure 7:**
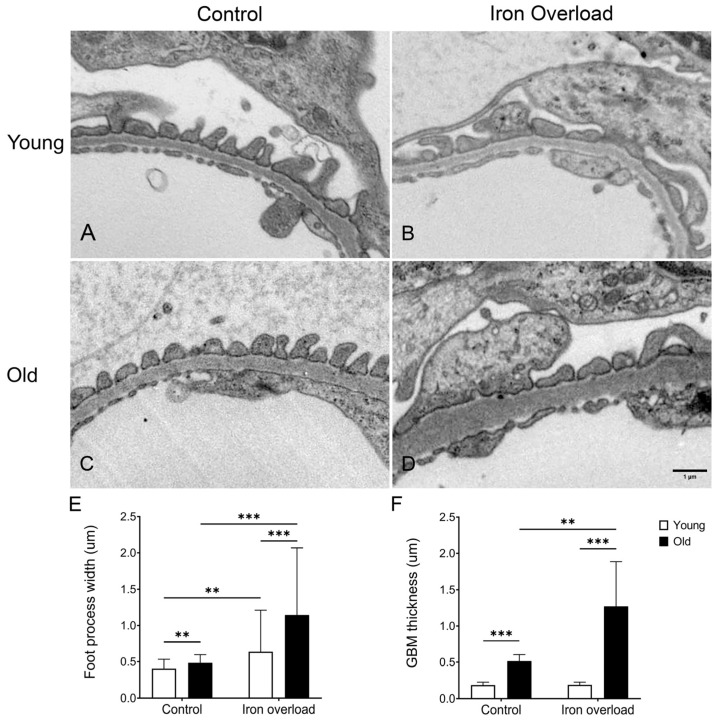
Structural changes in the GBM and foot process width induced by iron overload. Representative TEM images of glomerular structures in the young (**A**) and old control groups (**C**). Foot process widening was observed in the young iron overload group (**B**), with the old iron overload group showing more pronounced GBM thickening and foot process widening (**D**). Measurement of the GBM thickness from TEM (**E**). Quantification of the foot process width from TEM (**F**). The difference was significant at ** *p* < 0.01 and *** *p* < 0.001. Scale bar = 1 μm.

The effects of iron overload on the GBM thickness and foot process width in the kidney were evaluated using TEM (Figure 7). The GBM serves as a filtration barrier in the glomerulus and is located between endothelial cells and podocytes [16]. Normal GBM thickness and foot process structures were observed in the young control group (Figure 7A). However, foot process widening was noted in the young iron overload group (Figure 7B). Compared with the young control group, increased GBM thickness was observed in the old control group (Figure 7C). Moreover, pronounced GBM thickening and foot process widening were observed in the old iron overload group (Figure 7D). Therefore, the foot process width was measured and analyzed (Figure 7E). The foot process width increased from 0.40 ± 0.13 µm in the young control group to 0.49 ± 0.11 µm in the old control group (*p* < 0.01). Similarly, a significant increase in the foot process width was observed in the young iron overload group (0.64 ± 0.57 µm) compared with the old iron overload group (1.14 ± 0.93 µm, *p* < 0.001). The foot process width in the young iron overload group was greater than that in the young control group (*p* < 0.01). Moreover, the old iron overload group exhibited a significant increase in the foot process width compared with the old control group (*p* < 0.001).

Previous studies have reported that the GBM thickness increases with age-related changes [51,52]. The TEM images showed increased GBM thickness in the old control and iron overload groups (Figure 7C,D). To investigate this phenomenon in greater detail, the GBM thickness was measured (Figure 7F). The results showed an increase in the GBM thickness from the young control group (0.18 ± 0.04 µm) to the old control group (0.50 ± 0.07 µm, *p* < 0.001). A significant difference was also observed between the young iron overload group (0.19 ± 0.04 µm) and old iron overload group (1.27 ± 0.62 µm) (*p* < 0.001). Interestingly, the GBM thickness was significantly greater in the old iron overload group than in the old control group (*p* < 0.001).

## 4. Discussion

In the present study, we examined the effects of excessive iron on the kidneys of young and old mice using iron dextran. Although iron is essential for many biological processes, excess iron is toxic as it generates free radicals [53,54]. Therefore, the role of iron in the pathophysiology of various diseases needs to be considered.

Following the establishment of the iron overload model, the analysis of the iron test results revealed significantly increased serum iron levels, TIBC, and TF saturation in the iron overload groups compared with the control groups, indicating a higher iron concentration in the bloodstream. These findings suggest that most TF, which plays a crucial role in iron transport in the blood, is bound to iron. Conversely, the observed decrease in UIBC in the iron overload groups suggests that most TF is bound to iron, reducing the amount of free TF. These findings suggest that iron overload results in an increased iron concentration in the bloodstream, with no notable variations observed across different age groups.

Serum creatinine levels, an indicator of kidney function, were significantly elevated in the iron overload groups compared with the control groups. The old iron overload group showed higher serum creatinine levels, indicating that aging worsens iron overload-induced kidney dysfunction. These findings indicate a potential age-related vulnerability to kidney injury under conditions of iron overload.

In this model, the urinary iron levels were measured to determine whether excessive iron was filtered into the urine. Under normal physiological conditions, circulating iron is filtered by the glomerulus and almost completely reabsorbed by the tubular epithelium to prevent iron loss through urine [19,24,55,56]. According to previous studies, damage to the glomerular barrier and tubules increases urinary iron excretion by enhancing filtration and reducing reabsorption [26,55]. Urinary iron excretion was increased in the iron overload groups compared with the control groups. The old iron overload group exhibited significantly higher urinary iron excretion than the young iron overload group, suggesting functional impairment in filtration and reabsorption.

Furthermore, albuminuria was significantly higher in the old groups than in the young groups, with the old iron overload group exhibiting the highest levels of albuminuria. This finding may result from the increased permeability of the GBM because of aging, leading to enhanced urinary protein excretion [44,45,57]. Furthermore, aging may exacerbate iron-induced glomerular and tubular damage, resulting in increased protein leakage into the urine.

In our acute iron overload model, iron was confirmed by Perls’ Prussian blue staining and detected in both the glomerulus and interstitium. A similar pattern of glomerular iron deposition has been reported in hemolysis-induced models [58]; however, in our model, iron was also observed in the interstitium. In particular, iron deposition was significantly higher in the old iron overload group than in the young iron overload group, indicating that aging may be a contributing factor to increased iron accumulation in the kidney. The results of periodic acid–Schiff staining showed that iron overload caused glomerular damage, which was more severe in aged mice, suggesting a higher susceptibility to iron-induced injury with aging. Renal fibrosis was more pronounced in aged mice, especially under iron overload, suggesting that aging may increase susceptibility to iron-induced renal injury.

The glomerulus is a capillary network lined by endothelial cells, with central mesangial cells and podocytes covering the capillaries [14]. In the present study, iron accumulation was confirmed in mesangial cells under iron overload, with the old iron overload group showing a greater deposition. TEM analysis identified mesangial cells containing intracellular iron deposits, which is consistent with previous reports [28,29]. Furthermore, we observed that aging appears to exacerbate iron deposition, leading to an increase in the number of electron-dense deposits and phagolysosomes. Mesangial cells play a phagocytic role in clearing trapped proteins and immune complexes from the GBM [59,60]. Mesangial cells appear to actively take up excess iron, as indicated by the presence of iron-laden phagolysosomes.

Endothelial cells constitute the first barrier that regulates the passage of blood components from the capillary lumen to Bowman’s space [14]. Previous studies have reported iron accumulation in glomerular endothelial cells under iron overload conditions [28]. Our findings are consistent with these reports. However, we observed that iron deposition was more pronounced in the old iron overload group than in the young iron overload group. This finding indicates that iron can freely pass through the glomerular capillaries and reach the mesangial cells, leading to iron accumulation.

Iron overload significantly impacts the glomerular structure, extending beyond the mesangial and endothelial cells to affect the GBM and podocyte foot processes. The podocytes and GBM work together to maintain the glomerular filtration barrier, thereby preventing proteinuria. The slit diaphragm, a specialized junction between the podocyte foot processes, regulates filtration, and GBM or slit diaphragm defects can cause proteinuria [61,62].

Our findings demonstrate that iron overload accelerates age-related glomerular changes, particularly GBM thickening and foot process widening, as observed in the TEM analysis. Although previous studies have shown that aging alone contributes to GBM thickening [33,52], our results indicate that excessive iron further exacerbates these structural changes. Podocyte injury is a key pathological feature in focal segmental glomerulosclerosis and diabetic nephropathy, leading to foot process effacement, filtration barrier disruption, and progressive structural collapse of the glomerular architecture [61,63]. Similarly, in the present study, iron-overload-induced podocyte injury was found. In particular, the loss of interdigitating foot processes in aged mice may have compromised the filtration barrier, potentially resulting in increased albumin leakage.

We demonstrated that iron overload affects both the glomerulus and interstitium, with prominent glomerular involvement confirmed by TEM, and aging further exacerbates kidney injury. Our findings highlight the interaction between aging and iron toxicity, suggesting that older kidneys may be more susceptible to structural and functional impairment under iron overload conditions. However, this study has certain limitations. Notably, the dark brown color of the serum in the iron overload groups may have affected the colorimetric creatinine assay. Therefore, serum creatinine results should be interpreted with consideration of this potential limitation. In addition, although we examined the effects of iron overload and aging, the precise mechanisms underlying the increased vulnerability of aged kidneys remain unclear. Further studies are needed to better understand these mechanisms, which may provide insights for future therapeutic strategies.

## 5. Conclusions

In this study, iron overload induced both functional and structural kidney alterations, which appeared to be more prominent in aged mice. The old iron overload group showed relatively higher levels of albuminuria, serum creatinine, and urinary iron excretion, along with more evident histological changes such as GBM thickening and podocyte foot process widening. These findings suggest that aging may increase the susceptibility to iron-induced renal injury. 

## Data Availability

The original contributions presented in this study are included in the article. Further inquiries can be directed to the corresponding author.

## Abbreviations

GBM	glomerular basement membrane
PAS	periodic acid–Schiff
TBI	transferrin-bound iron
TEM	transmission electron microscopy
TF	transferrin
TIBC	total iron-binding capacity
UIBC	unsaturated iron-binding capacity
